# Comparison of two different topical 0.05% cyclosporine A formulations on ocular surface outcomes following pterygium excision with conjunctival autograft

**DOI:** 10.1097/MD.0000000000047941

**Published:** 2026-03-13

**Authors:** Soyoung Ryu, Ha Young Chung, Kookyoung Kim, Hungwon Tchah, Kyungmin Koh

**Affiliations:** a Department of Ophthalmology, Kim’s Eye Hospital, Konyang University College of Medicine, Seoul, Republic of Korea.

**Keywords:** conjunctival autograft, cyclosporine, pterygium, recurrence, tear meniscus height

## Abstract

A topical 0.05% cyclosporine A (CsA) nanoemulsion formulation was developed to improve tolerability and efficacy. However, its role in preventing recurrence and improving ocular surface parameters after pterygium surgery remains unclear. This study aimed to compare the efficacy and safety of 2 different topical 0.05% CsA formulations following pterygium excision with conjunctival autograft. This retrospective comparative study included patients who underwent pterygium excision with conjunctival autograft using fibrin glue by a single surgeon between January 2021 and June 2024. Postoperatively, patients were administered either 0.05% CsA emulsion (CsA-E; *Restasis^®^*, Allergan Inc., Irvine) or 0.05% CsA nanoemulsion (CsA-NE; *Cyporin N^®^*, Taejoon Pharm Co., Ltd., Seoul, Republic of Korea) twice daily for 12 months. The primary outcome was the recurrence rate of pterygium at 12 months postoperative. Secondary outcomes included uncorrected (UDVA) and corrected (CDVA) distance visual acuity, tear meniscus height (TMH), and adverse events. A total of 202 eyes (202 patients) was analyzed: 96 in the CsA-E group (60 males, 36 females; mean age, 65.6 ± 8.2 years) and 106 in the CsA-NE group (64 males, 42 females; mean age, 65.2 ± 8.4 years). There were no significant differences in baseline characteristics between the 2 groups. The recurrence rate was 8.3% in the CsA-E group and 3.8% in the CsA-NE group, with no statistically significant difference (*P* = .235). Postoperative VA outcomes were comparable between the groups; the median CDVA was 0.04 logMAR in both groups (*P* = .175). Notably, TMH values in both groups significantly decreased compared to baseline (both *P* <.001), normalizing to within the physiological range, with no significant difference in the magnitude of reduction between the groups (*P* = .641). No severe adverse events were reported in either group. Both CsA formulations were effective in preventing pterygium recurrence with comparable safety profiles. Additionally, both groups achieved excellent and comparable postoperative VA.

## 1. Introduction

Pterygium is a conjunctival overgrowth onto the cornea that can cause visual disturbance, ocular motility restriction, chronic inflammation, and cosmetic concerns.^[1]^ Pterygium is a common ocular surface disease, and although its clinical characteristics and treatment strategies have been studied extensively, several issues are still unresolved.^[2]^ Pterygium has been shown to disturb tear film stability, and its physical size may further influence tear meniscus height (TMH), an important indicator of tear volume and ocular surface condition.^[3]^ Surgical excision is the standard treatment, but recurrence remains a major limitation.^[4]^ Conjunctival autograft is the preferred technique to reduce recurrence, and it can be fixed either with sutures or fibrin glue (FG). While sutures may prolong surgery and cause discomfort or complications, FG shortens the procedure and improves postoperative comfort.^[5]^

Cyclosporine A (CsA) is a calcineurin inhibitor with anti-inflammatory effects, suppressing T-helper cells, interleukin synthesis, and vascular endothelial growth factor.^[6,7]^ CsA has proven safety and efficacy in the treatment of dry eye syndrome.^[8]^ Studies have shown that 0.05% CsA suppresses fibroblast proliferation in both pterygium and normal conjunctiva.^[9,10]^ A meta-analysis reported that conjunctival autograft with 0.05% topical CsA resulted in a reduced recurrence rate.^[11]^

Since CsA is insoluble in water, 0.05% CsA eye drops (CsA-E; *Restasis*^®^, Allergan Inc., Irvine) are formulated as an emulsion, with castor oil added to enhance solubility.^[12]^ A common adverse effect of the emulsion formulation is a burning sensation, which often leads to reduced patient compliance.^[13]^ To overcome this limitation, a 0.05% CsA nanoemulsion (CsA-NE; *Cyporin N*^®^, Taejoon Pharm Co., Ltd., Seoul, Republic of Korea) was developed, characterized by its transparent appearance and uniform particle size.^[14]^ It prolongs precorneal residence time, ocular bioavailability, and anti-inflammatory efficacy, and is increasingly applied in dry eye treatment.^[13,15,16]^ However, comparative data on ocular surface outcomes between CsA-E and NE following pterygium excision with conjunctival autograft remain limited.

Therefore, we investigated whether long-term postoperative use of the 2 CsA formulations influences not only the recurrence of pterygium but also changes in ocular surface outcomes, and whether there are differences in their therapeutic efficacy.

## 2. Methods

### 2.1. Study design and objectives

This single-center, retrospective, comparative study was conducted in accordance with the tenets of the Declaration of Helsinki and was approved by the Institutional Review Board of the Kim’s Eye Hospital, Seoul, Republic of Korea (approval number: 2025-03-004). Owing to the retrospective design and use of de-identified patient data, the requirement for written informed consent was waived by the Institutional Review Board. Moreover, this study did not include any personal information that could identify the patients, and the data was analyzed anonymously. This retrospective study reviewed patients who underwent pterygium excision with conjunctival autograft using FG performed by a single surgeon (KK) between January 2021 and June 2024 at Kim’s Eye Hospital, Seoul, Republic of Korea. To minimize the influence of individual patients, only the first operated eye was included in cases of bilateral surgery.

Patients were divided into 2 groups according to the postoperative CsA formulation: CsA-E and CsA-NE. The study evaluated pterygium recurrence within one year after surgery, the incidence of adverse effects related to one year of topical CsA use, and changes in uncorrected distant visual acuity (UDVA), corrected distant visual acuity (CDVA), steep keratometry (K), flat K, and TMH at the one-year postoperative visit. Comparative analyses were performed to determine whether significant differences were present between the 2 treatment groups.

### 2.2. Criteria for inclusion and exclusion

Patients were eligible for inclusion if they had a primary pterygium that required surgical excision, underwent conjunctival autografting with FG, received postoperative topical 0.05% CsA (either CsA-E or CsA-NE), and completed at least 12 months of follow-up. Patients with recurrent pterygium, previous ocular surgery or trauma in the study eye, concomitant ocular surface disorders such as severe dry eye, ocular cicatricial pemphigoid, or limbal stem cell deficiency, active ocular infection or inflammation, systemic use of immunosuppressive agents, or incomplete medical records were excluded from the analysis. Patients with a prior history of ocular diseases or surgeries capable of influencing measurements, individuals with systemic conditions such as diabetes, autoimmune diseases, and other factors that could impact post-surgery parameters were excluded. Additionally, patients with incomplete data or those who were unsuitable for preoperative or postoperative assessments were excluded.

### 2.3. Topical cyclosporine A formulations

CsA-E is an anionic oil-in-water emulsion without preservatives, in which CsA is dissolved in castor oil with polysorbate as an emulsifier.^[17]^ Because CsA-E is an anisotropic complex emulsion, contact with the tear film may cause surfactant release.^[18]^ In addition, uneven particle size can lead to aggregation or cream formation, thereby reducing long-term physical and chemical stability. As a result, adverse effects such as blurred vision, conjunctival congestion, and burning sensations after instillation have been frequently reported.^[19]^ Furthermore, since CsA exists in multiple phases, drug delivery to the ocular tissue may be insufficient.^[13]^

In contrast, CsA-NE is NE CsA products developed using a self-nanoemulsifying drug delivery system.^[13]^ Self-nanoemulsifying drug delivery system is an anhydrous homogeneous mixture of oil, drug, surfactants, and co-surfactants that forms a transparent NE with uniform particle size. This structure provides excellent physical and chemical stability.^[17,20,21]^

### 2.4. Preoperative assessment

The following preoperative evaluations were performed for all patients: slit lamp examination, fundus examination, UDVA and CDVA measured at 6 m using the ETDRS chart. TMH were assessed using a Keratograph 5M (Oculus, Wetzlar, Germany).^[22]^ Slit-lamp cameras were used for imaging the participants, after which the pterygium size were measured. Flat and steep K measured using anterior-segment optical coherence tomography device (ANTERION, Heidelberg Engineering GmbH, Germany). Anterior segment photographs were taken using a slit lamp (BQ 900, Haag-Streit AG, Köniz, Switzerland) to evaluate preoperative pterygium, including horizontal extension from the limbus to the apex and the area of corneal involvement.^[23]^ All measurements were performed by the same examiner (HYC).

### 2.5. Surgical procedure

All surgical procedures were performed by a single surgeon (KK). Subtenon anesthesia was administered with 2% lidocaine, followed by wide excision of the pterygium and subconjunctival fibrovascular tissue. The pterygium head was carefully dissected from the cornea, and the remaining body was removed with conjunctival scissors. In every case, a free conjunctival autograft was harvested from the superotemporal conjunctiva, including a 1.0-mm limbal margin, and secured with FG (Greenplast®, SK Plasma, Seongnam, Republic of Korea). Postoperatively, all patients received topical antibiotics (Gatifloxacin 0.3%; Gatiflo^®^, Samil Co, Ltd., Seoul, Republic of Korea) and steroids (Fluorometholone 0.1%; Fulleylone^®^, Binex Co., Busan, Republic of Korea) 4 times a day for 2 weeks. These medications were discontinued without tapering. Subsequently, 0.05% CsA was initiated twice daily and maintained for 12 months.

### 2.6. Statistical analysis

All statistical analyses were performed using SPSS software (version 26.0; IBM Corp., Armonk). The normality of continuous variables was assessed using the Shapiro–Wilk test. Continuous variables are presented as mean ± standard deviation for normally distributed variables and as median (interquartile range, IQR) for non-normally distributed variables. Categorical variables are presented as number (%). For between-group comparisons at baseline and for postoperative absolute values, the Student *t* test or Welch *t*-test was used for normally distributed variables, while the Mann–Whitney *U* test was used for non-normally distributed variables. Categorical variables were compared using the χ^2^ test or Fisher exact test, as appropriate. Within-group comparisons between preoperative and postoperative values were performed using the Wilcoxon signed-rank test. Between-group comparisons of changes from baseline to 12 months postoperatively were conducted using the Mann–Whitney *U* test. A *P*-value <.05 was considered statistically significant.

## 3. Results

A total of 202 eyes from 202 patients were included in the analysis. The CsA-E group comprised 96 eyes (60 males, 36 females; right eyes 41, left eyes 55) with a mean age of 65.6 ± 8.2 years (range, 44–84), and the CsA-NE group comprised 106 eyes (64 males, 42 females; right eyes 44, left eyes 62) with a mean age of 65.2 ± 8.4 years (range, 38–85). Baseline characteristics were comparable between groups (Table 1). There were no significant differences in sex distribution (*P* = .869), laterality (*P* = .976), or age (65.6 ± 8.2 vs 65.2 ± 8.4 years, *P* = .710).

**Table 1:** Baseline characteristics of patients in the 2 treatment groups.

Parameter	CsA-E		CsA-NE		*P*-value
Patients/Eyes, n		96/96		106/106	N/A
Female		36 (37.5%)		42 (39.6%)	.869*
Left eye		55 (57.3%)		62 (58.5%)	.976*
Age (yr)		65.6 ± 8.2 (44–84)		65.2 ± 8.4 (38–85)	.710†
UDVA (LogMAR)		0.22 (0.10–0.40)		0.22 (0.10–0.40)	.783‡
CDVA (LogMAR)		0.04 (0.00–0.10)		0.04 (0.00–0.10)	.362‡
Flat keratometry (D)		42.05 (40.65–43.12)		42.75 (41.20–43.88)	.078‡
Steep keratometry (D)		43.55 (42.60–45.08)		44.25 (42.62–45.70)	.218‡
Tear meniscus height (mm)		0.34 (0.25–0.47)		0.33 (0.20–0.45)	.212‡
Pterygium size (mm)		3.00 (2.00–4.00)		3.00 (2.00–3.00)	.198‡

Values are presented as mean ± standard deviation (SD) for normally distributed variables and median (interquartile range, IQR) for non-normally distributed variables. Number (%) is used for categorical variables. Visual acuity was expressed as logMAR (logarithm of the minimum angle of resolution).

CDVA = corrected distant visual acuity, CsA-E = 0.05% cyclosporine A emulsion (*Restasis*^*®*^, Allergan Inc., Irvine), CsA-NE = 0.05% cyclosporine A nanoemulsion (*Cyporin N*^*®*^, Taejoon Pharm Co., Ltd., Seoul, Republic of Korea), D = dioptre, N/A = not applicable, UDVA = uncorrected distant visual acuity.

† Student *t* test.

‡ Mann–Whitney *U* test.

* Pearson χ^2^ test.

Baseline UDVA and CDVA were similar between the 2 groups (median UDVA: 0.22 [IQR, 0.10–0.40] vs 0.22 [IQR, 0.10–0.40] logarithm of the minimum angle of resolution (logMAR), *P* = .783; median CDVA: 0.05 [IQR, 0.00–0.10] vs 0.05 [IQR, 0.00–0.10] logMAR, *P* = .362). Baseline keratometric values and TMH did not differ significantly (median Flat K: 42.05 [40.65–43.12] vs 42.75 [41.20–43.88] D, *P* = .078; median Steep K: 43.55 [42.60–45.08] vs 44.25 [42.62–45.70] D, *P* = .218; median TMH: 0.34 [0.25–0.47] vs 0.33 [0.20–0.45] mm, *P* = .212).

At 12 months postoperatively, there were no significant differences between the CsA-E and CsA-NE groups in UDVA or CDVA (median UDVA: 0.22 [0.04–0.40] vs 0.15 [0.10–0.30] logMAR, *P* = .923; median CDVA: 0.04 [0.00–0.15] vs 0.04 [0.00–0.09] logMAR, *P* = .175). Flat and steep K values were also comparable (median Flat K: 42.50 [41.40–43.45] vs 42.90 [41.70–43.90] D, *P* = .223; median Steep K: 43.50 [42.60–44.52] vs 43.95 [42.82–45.08] D, *P* = .116). However, median TMH was significantly lower in the CsA-NE group (0.28 [0.19–0.40] vs 0.21 [0.16–0.31] mm, *P* = .012). Recurrence occurred in 8 of 96 eyes (8.3%) in the CsA-E group and 4 of 106 eyes (3.8%) in the CsA-NE group, with no statistically significant difference (*P* = .235) (Table 2).

**Table 2:** Twelve-month clinical outcomes following pterygium excision with conjunctival autograft and postoperative topical 0.05% cyclosporine A.

Parameter	CsA-E	CsA-NE	*P*-value
UDVA (LogMAR)	0.22 (0.04–0.40)	0.15 (0.10–0.30)	.978†
CDVA (LogMAR)	0.04 (0.00–0.15)	0.04 (0.00–0.09)	.175†
Flat keratometry (D)	42.50 (41.40–43.45)	42.90 (41.70–43.90)	.223†
Steep keratometry (D)	43.50 (42.60–44.52)	43.95 (42.82–45.08)	.116†
Tear meniscus height (mm)	0.28 (0.19–0.40)	0.21 (0.16–0.31)	**.012** * ^,^ †
Recurrence	8/96 (8.3%)	4/106 (3.8%)	.235‡

Values are presented as median (interquartile range) for continuous variables and number (%) for categorical variables.

CDVA = corrected distant visual acuity, CsA-E = 0.05% cyclosporine A emulsion (*Restasis*^*®*^, Allergan Inc., Irvine), CsA-NE = 0.05% cyclosporine A nanoemulsion (*Cyporin N*^*®*^, Taejoon Pharm Co., Ltd., Seoul, Republic of Korea), D = diopter, UDVA = uncorrected distant visual acuity.

† Mann–Whitney *U* test.

‡ Fisher exact test.

* Bold values indicate statistical significance (*P* <.05).

Within-group analysis showed a significant reduction in TMH from baseline to 12 months in both groups (both *P* <.001). Between-group comparisons of changes revealed that TMH, UDVA, CDVA, and steep K were not significantly different between the groups. Regarding corneal curvature, only the change in flat K showed a significant difference between the 2 groups (*P* <.001), with the CsA-E group showing an increase compared to the CsA-NE group (Table 3).

**Table 3:** Comparison of clinical parameter changes from baseline between the groups at 12 mo.

Parameter	Change in CsA-E		Change in CsA-NE		*P*-value‡
		*P*-value†		*P*-value†	
UDVA	0.00 (−0.10 to 0.00)	.371	0.00 (−0.15 to 0.09)	.325	.838
CDVA	0.00 (−0.01 to 0.05)	.898	0.00 (−0.05 to 0.05)	.680	.682
Flat K (D)	+0.40 (+0.30 to +0.60)	**<.001** *	0.00 (−0.39 to +0.80)	.117	**<.001** *
Steep K (D)	−0.17 (−0.33 to +0.30)	.329	0.00 (−0.59 to +0.30)	.062	.918
TMH (mm)	−0.06 (−0.08 to −0.05)	**<.001** *	−0.05 (−0.22 to 0.00)	**<.001** *	.641

Values are presented as median (interquartile range). Change was calculated as the value at 12 mo minus the baseline value. Visual acuity was expressed as logMAR (logarithm of the minimum angle of resolution).

CDVA = corrected distant visual acuity, CsA-E = 0.05% cyclosporine A emulsion (*Restasis*^*®*^, Allergan Inc., Irvine), CsA-NE = 0.05% cyclosporine A nanoemulsion (*Cyporin N*^*®*^, Taejoon Pharm Co., Ltd., Seoul, Republic of Korea), D = diopter, K = keratometry, POD 1Y = 1 yr postoperatively, TMH = tear meniscus height, UDVA = uncorrected distant visual acuity.

† Intragroup analysis (Wilcoxon signed-rank test).

‡ Intergroup analysis (Mann–Whitney *U* test).

* Bold values indicate statistical significance (*P* <.05).

No intraoperative complications were reported, and no postoperative complications other than recurrence were observed during the follow-up period in either group.

## 4. Discussion

This study compared the 12-month postoperative clinical outcomes of 2 different topical 0.05% CsA formulations following pterygium excision with conjunctival autograft. Both formulations demonstrated comparable efficacy in preventing recurrence. Postoperative UDVA and CDVA remained stable in both groups with no significant intergroup differences. TMH significantly decreased in both groups, reflecting the anatomic normalization of tear drainage following pterygium removal.

Pterygium surgery with conjunctival autografting, in which a free conjunctival graft is used to cover the scleral bed after excision, is regarded as more technically demanding and time-consuming but consistently achieves lower recurrence rates than bare sclera excision.^[24]^ The graft may be secured with either sutures or tissue adhesives, with common drawbacks including transient ocular discomfort and, less frequently, graft displacement. Persistence of Tenon tissue within the graft has been identified as a risk factor for fibroblast proliferation and recurrence, and Tenon-free grafts are therefore recommended.^[25]^ Multiple studies have further demonstrated that conjunctival autografting is superior to the bare sclera approach in reducing recurrence of both primary and recurrent pterygia.^[26]^ FG has been used as an alternative to sutures in pterygium surgery, providing shorter operative time and lower recurrence but limited by higher cost and risks of infection or graft-related complications.^[27]^ Reported recurrence rates were 5.3% with FG versus 13.5% with sutures.^[28]^ A comparative trial of 60 cases showed FG to be the most efficient method, with the shortest surgical time, least discomfort, and lowest recurrence rate.^[29]^

Given that the surgical technique was identical in both groups, the comparable recurrence rates (8.3% vs 3.8%, *P* = .235) suggest that the 0.05% CsA-NE formulation possesses comparable efficacy to the conventional emulsion in preventing pterygium recurrence. These findings indicate that both formulations are effective adjunctive therapies for postoperative management.

Several studies have investigated the role of postoperative topical CsA as an adjuvant therapy in reducing pterygium recurrence. In a comparative analysis of 31 patients who underwent bilateral bare sclera excision, recurrence was observed in 12.9% of eyes treated with 0.05% CsA, compared with 45.2% in the control group, corresponding to a 7.37-fold higher recurrence risk without CsA.^[30]^ Another study reported a recurrence rate of 20.6% in the CsA group, which was lower than that in controls.^[31]^ Furthermore, a randomized controlled trial involving 36 eyes treated with bare sclera excision found that postoperative 0.05% CsA resulted in recurrence in 22.2% of cases, which was approximately half of the recurrence rate in the control group.^[32]^ A recent meta-analysis of 12 studies (893 eyes) demonstrated that adjuvant topical 0.05% CsA significantly reduces pterygium recurrence rates compared with excision surgery alone. However, this benefit was most evident after bare sclera and conjunctival flap rotation techniques, while no significant effect was observed with rotational or limbal conjunctival autografts.^[1]^ In the present study, all patients underwent surgery with limbal conjunctival autograft and FG, which may explain the lack of significant differences in recurrence between the formulations.

Visual outcomes have been variably reported following pterygium surgery. Several studies demonstrated improvement in UDVA or CDVA after excision with different techniques, including bare sclera, conjunctival autograft, limbal stem cell autograft, and amniotic membrane graft.^[23,33,34]^ However, other studies reported no significant changes, particularly after conjunctival autograft procedures.^[23,35]^

In a previous study, no significant change in TMH was observed up to 6 months after pterygium excision with conjunctival autograft.^[22]^ In the present study, TMH significantly decreased in both groups at 12 months, consistent with persistent postoperative tear film instability. Interestingly, although absolute TMH values were lower in the CsA-NE group, the degree of reduction did not significantly differ between groups, suggesting similar effects of both formulations on tear film homeostasis.

Previous studies have reported that the mean normal TMH in healthy eyes typically ranges from 0.20 to 0.30 mm.^[36–38]^ In the present study, TMH values in both groups effectively normalized to within this physiological range following pterygium surgery and topical CsA administration, with no significant difference in the magnitude of reduction between the 2 groups (*P* = .641). This suggests that both CsA formulations are equally effective in restoring tear film dynamics after the removal of the pterygium’s mechanical obstruction.^[15,39]^

The slightly lower absolute postoperative TMH observed in the CsA-NE group may be attributed to its formulation characteristics. The nanometer-sized droplets provide a larger surface area and enhanced tissue penetration compared to conventional emulsions, facilitating rapid absorption and minimizing the duration of ocular surface irritation.^[39–41]^ Overall, our findings indicate that both CsA formulations are comparable in their ability to facilitate the anatomic and physiological normalization of the ocular surface postoperatively.

This study has several limitations. First, its retrospective design without randomization may have introduced potential bias despite strict inclusion and exclusion criteria. Specifically, strict matching for pterygium size, morphology, or conjunctival autograft size was not performed. However, baseline clinical characteristics were comparable between the groups, and all surgeries were performed by a single experienced surgeon using a standardized technique. Second, the follow-up period was limited to 12 months. Since the therapeutic effects of CsA are time-dependent and pterygium recurrence may occur beyond 12 months, longer follow-up would allow better evaluation of long-term outcomes. Third, preoperative pterygium severity was assessed primarily by horizontal length, which may not fully capture disease activity.^[42]^ The standard grading system of pterygium was not applied, which may have affected the assessment of recurrence rates.^[43,44]^ Future studies should incorporate standardized grading systems and lesion depth measurements to improve comparability. Fourth, formal compliance surveys were not performed due to the retrospective nature of the study. However, considering the high cost of the medication, the continuity of prescriptions serves as an indirect indicator of compliance. A review of medical records revealed no instances of treatment discontinuation caused by ocular intolerance or adverse events in either group.

In conclusion, after pterygium excision and conjunctival autografting using FG, our study demonstrated that 0.05% CsA-NE provides comparable efficacy to the conventional 0.05% CsA emulsion in preventing pterygium recurrence. Both formulations were associated with equivalent visual outcomes and successfully led to the anatomic normalization of tear film dynamics. These findings suggest that the 0.05% CsA-NE is a viable and effective therapeutic alternative to the conventional emulsion for the long-term postoperative management of pterygium.

## Author contributions

**Conceptualization:** Soyoung Ryu, Hungwon Tchah, Kyungmin Koh.

**Data curation:** Soyoung Ryu, Ha Young Chung.

**Formal analysis:** Ha Young Chung.

**Investigation:** Hungwon Tchah.

**Methodology:** Soyoung Ryu, Ha Young Chung.

**Resources:** Kookyoung Kim, Kyungmin Koh.

**Software:** Kookyoung Kim.

**Supervision:** Hungwon Tchah, Kyungmin Koh.

**Validation:** Kyungmin Koh.

**Visualization:** Kookyoung Kim.

**Writing – original draft:** Soyoung Ryu, Kyungmin Koh.

**Writing – review & editing:** Hungwon Tchah, Kyungmin Koh.
